# An Aggregated-Based Deep Learning Method for Leukemic B-lymphoblast Classification

**DOI:** 10.3390/diagnostics10121064

**Published:** 2020-12-08

**Authors:** Payam Hosseinzadeh Kasani, Sang-Won Park, Jae-Won Jang

**Affiliations:** 1 Department of Neurology, Kangwon National University Hospital, Chuncheon 24289, Korea; Payam.kassani@kangwon.ac.kr (P.H.K.); chicwon229@kangwon.ac.kr (S.-W.P.); 2 Department of Medical Bigdata Convergence, Kangwon National University, Chuncheon 24289, Korea; 3 College of Medicine, Kangwon National University, Chuncheon 24289, Korea

**Keywords:** acute lymphoblastic leukemia, deep learning, transfer learning, computer-aided diagnosis

## Abstract

Leukemia is a cancer of blood cells in the bone marrow that affects both children and adolescents. The rapid growth of unusual lymphocyte cells leads to bone marrow failure, which may slow down the production of new blood cells, and hence increases patient morbidity and mortality. Age is a crucial clinical factor in leukemia diagnosis, since if leukemia is diagnosed in the early stages, it is highly curable. Incidence is increasing globally, as around 412,000 people worldwide are likely to be diagnosed with some type of leukemia, of which acute lymphoblastic leukemia accounts for approximately 12% of all leukemia cases worldwide. Thus, the reliable and accurate detection of normal and malignant cells is of major interest. Automatic detection with computer-aided diagnosis (CAD) models can assist medics, and can be beneficial for the early detection of leukemia. In this paper, a single center study, we aimed to build an aggregated deep learning model for Leukemic B-lymphoblast classification. To make a reliable and accurate deep learner, data augmentation techniques were applied to tackle the limited dataset size, and a transfer learning strategy was employed to accelerate the learning process, and further improve the performance of the proposed network. The results show that our proposed approach was able to fuse features extracted from the best deep learning models, and outperformed individual networks with a test accuracy of 96.58% in Leukemic B-lymphoblast diagnosis.

## 1. Introduction

### 1.1. Background

**Figure 1:**
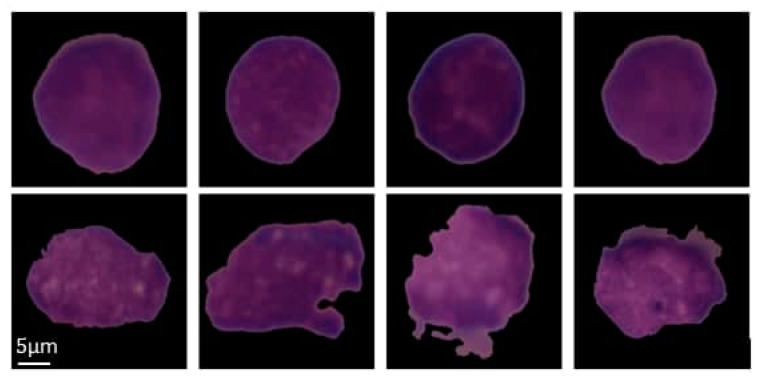
Examples of images from the ISBI 2019 challenge: Normal B cells (top row), leukemic B-lymphoblast cells (bottom row). The dataset used in this study was provided by SBILab [10], and was stain-normalized as in [11,12].

Leukemia is a cancer of blood cells in the bone marrow that affects both children and adolescents. Leukemia can be divided into acute or chronic categories, based on how quickly it progresses. Acute leukemia can be subdivided into acute lymphoblastic leukemia (ALL) and acute myeloid leukemia (AML), based on the type of blood cell that is affected [1]. The most common type of leukemia in children is ALL. In ALL, lymphocytes, a type of white blood cell (WBC), in the bone marrow do not mature properly into normal cells and reproduce out of control. By invading the blood cells, cancerous cells can spread to different organs and affect the whole body [2]. The rapid growth of unusual cells can lead to bone marrow failure, which can be lethal if not diagnosed until the later stages of the disease or if the treatment process is delayed. Age is a crucial clinical factor that should be taken into account in ALL diagnosis. The risk then reduces at a slow pace until the mid-20 s, and begins to increase again after age 50. Overall, about four of every 10 cases of ALL are in adults. According to the data provided by [3], In 2019, about 6150 new cases of Leukemia of type ALL were diagnosed, and about 1520 patients died of ALL, including both children and adults in the United States. The risk of getting ALL is slightly higher in males than females, and higher in whites than African Americans. If ALL is diagnosed in its early stages, it is highly curable, and the survival rate of the patient increases. Based on the patient’s symptoms and risk-level, different treatment options are available, including chemotherapy, radiotherapy, anti-cancer medicine, or a combination of these. They help cure patients or reduce the symptoms of the disease. These treatment options significantly lengthen the lives of patients with ALL [4]. Selection of suitable treatment depends on several factors, such as the type and grade of the disease, age, and the general health of the patient. Additionally, stem cell transplantation for patients in remission is also an option [5]. The typical treatment for ALL is chemotherapy, which aims to prevent ALL from damaging the central nervous system (CNS) [1]. ALL examination is based on cell morphology, cytochemistry, cytogenetic, and molecular features. A variety of morphological features can be utilized to distinguish ALL and healthy cells from each other, such as: size and shape of the cell, size and shape of its nucleus, color distribution in the nucleus, texture of the nucleus, cytoplasm size, number of nucleoli in the nucleus, nucleus contour, and the boundary and condition of the cytoplasm [6]. The signs and symptoms of ALL may range from mild symptoms, such as fever, bleeding from the gums, fatigue, dizziness, and bone pain, to severe life-threatening symptoms, which demonstrate the extent of bone marrow involvement [7,8]. The examinations that are needed to confirm an ALL diagnosis are bone marrow aspiration and biopsy, complete blood count (CBC), and peripheral blood smear [9]. The nucleus-to-cytoplasm ratios in ALL and healthy cells are approximately 1/5 and 2/5, respectively. The healthy cells on a blood smear appear homogeneous and uniform, round-to-ovoid-shaped, small sized, and with a regular nuclear shape as demonstrated in Figure 1 (top row). ALL cells are heterogeneous in their shape and size. The shape of the ALL cells is elongated and atypical, with large amounts of chromatin (a mass of genetic material). The ALL lymphoblasts vary in size, and the shape of nuclei is very irregular, as demonstrated in Figure 1 (bottom row). Using CAD systems with the integration of advanced medical image processing and machine learning methods, we can provide real-time analysis and better decision-making tools.

### 1.2. Motivations and Contributions

In this paper, we present a novel ensemble method based on deep convolutional neural networks (DCNNs) to deal with Leukemic B-lymphoblast classification. The major contributions of this study are as follows:

1. The proposed aggregated model, based on deep learning architectures, achieves a better performance in terms of accuracy, precision, and recall for the ALL leukemia images. To the best of our knowledge, this is the first study toward the implementation of an aggregated model based on well-tuned deep learning algorithms for classification of ALL leukemia images applied to the ISBI 2019 challenge dataset.
2. To improve the prediction performance, we extensively investigated the effect of fine-tuning of the different architectures with different settings of optimizers and image normalization, in order to generate a more discriminative representation. With these changes, our proposed model achieved higher accuracy in ALL classification compared to other state-of-the-art deep learning-based architectures and machine learning models individually.
3. The performance of models trained by features extracted automatically using deep CNN models was compared to the performance of models trained by hand-crafted features, e.g., local binary pattern (LBP) descriptors. The obtained hand-crafted features were separately fed into the five leading machine learning classifiers using a k-fold cross-validation strategy. Classification results of the proposed ensemble model were compared with widely-used machine learning classifiers to measure the overall performance of the proposed method.

The remainder of the paper is organized as follows: A review of previous, related works is presented in Section 2. Section 3 presents the material and methods for pre-processing steps, data augmentation, convolutional neural network (CNN) architectures, and evaluation metrics. In Section 4, the experimental setup, and a discussion of the major findings of the proposed architecture, are presented. Finally, the conclusion and perspectives on future work are given in Section 5.

### 1.3. Related Studies

Over the last few years, machine learning methods have successfully solved the problem of finding hidden patterns or structures in data [13,14]. Several methods for ALL detection have been proposed in the literature. In a study made by Singhal et al. [10], automatic detection of ALL was proposed based on geometric features and local binary pattern (LBP) texture features. Then a support vector machine (SVM) classifier was used for classification of blast cases. The results showed that the LBP texture features performed slightly better than the shape features, with 89.72% and 88.79% classification accuracy, respectively.

Mohamed et al. [15] proposed an approach for diagnosing leukemia (ALL and AML) and Myeloma. First, they used different feature extraction methods such as scale invariant feature transform (SIFT), speeded up robust features (SURF), and oriented FAST and rotated BRIEF (ORB) approaches. For training a classifier, they fit computed features to different classifiers, such as K-nearest neighbors (KNN), SVM, and random forest (RF). Their proposed system achieved 94.3% accuracy using the RF classifier.

Another research project conducted by Yu et al. [16] proposed an automatic cell recognition system using a CNN. The results of several state-of-the-art CNNs, including ResNet50 [17]; InceptionV3 [18]; VGG16 [19]; VGG19 [19], and Xception [20] were combined to classify cells into their corresponding classes. Then a comparative evaluation of the prediction performance between DCNN models and several traditional models, such as KNN, SVM, logistic regression (LR), and decision tree (DT), was performed. The result showed that DCNN models achieved a prediction accuracy of 88.5%.

Honnalgere and Nayak [21] fine-tuned a VGG16 architecture for B-ALL white blood cancer classification. The authors employed a transfer learning strategy pretrained on the ImageNet dataset. In this study, a batch normalization technique helped to reduce the covariate shift and changes in distribution of the input data. Data augmentation methods, such as horizontal and vertical flips, alongside rotation by 90°, 180°, and 270°, were applied to increase the training sample size. The proposed method achieved a weighted precision of 91.70%, weighted recall of 91.75%, and weighted-F1score of 91.70%.

Marzahl et al. [22] presented an attention-based deep learning method using a ResNet-18 network, with an additional regression for classifying malignant acute lymphoblastic leukemia. Advanced data augmentation techniques, and transfer learning with adaptive learning rates, were also employed to improve the model performance and reduce the issue of overfitting on the small dataset. The proposed approach achieved a weighted F1 score of 87.46%.

Shah et al. [23] designed a custom-designed deep learning model by a combination of deep CNN and recurrent neural networks. The proposed ensemble model tackled the visual similarity between normal and malignant cells by extracting the spectral features using discrete cosine transform in conjunction with an recurrent neural network (RNN). The highest accuracy achieved by the proposed method was 86.6%. 

Pan et al. [24] proposed a neighborhood-correction algorithm using three steps. First, a pretrained residual network was fine-tuned and trained on data to produce initial labels and feature-maps for the testing phase. Then, a Fisher vector was constructed for each cell image based on feature maps extracted from the previous stage. As the last step, the initial predicted label of the test images was corrected based on weighted majority voting, and based on its most similar neighbors. The highest weighted F1-score achieved by the proposed method was 92.50%.

Finally, Mohapatra et al. [25] proposed an automated classifier method for acute leukemia detection in stained blood smear and bone marrow microscopic images. The results were obtained by using image segmentation, discriminative feature extraction, and an ensemble classifier on light microscopic images of stained blood films. The result showed that an ensemble of classifiers achieved an average 94.73% accuracy, with an average sensitivity and average specificity of greater than 90%, in comparison with other standard classifiers, e.g., naive Bayesian (NB), KNN, multilayer perceptron (MLP), radial basis functional network (RBFN), and SVM.

## 2. Materials and Methods 

In this section, several CNN architectures and their theoretical concepts are introduced. We start by presenting the pre-processing and data augmentation steps, and in Section 2.3 we present the DCNN models, transfer learning, local binary patterns, and evaluation metrics.

### 2.1. Data Pre-Processing

Image normalization is a process that changes the range of pixel intensity values by setting its mean to zero and variance to one. To do so, we used different methods of image normalization of the images to compare the performance of learners. First, we normalized the images by subtracting mean RGB values, computed by averaging over each image and the unit standard deviation, as suggested by Kawahara et al. [26]. Another normalization method used in this study was to subtract the mean of the RGB values of all images from the training set divided by its standard deviation [27]. We also normalized images using ImageNet mean subtraction as a pre-processing step. The ImageNet mean is a pre-computed constant derived from the ImageNet database [28].

Due to the black margin of each image as illustrated in Figure 1, we resized all images from the image center to the size of 380 × 380 pixels, using bicubic interpolation to ensure that each cell was located at the center, and to reduce the non-informative adjacent black background regions.

### 2.2. Data Augmentation

**Figure 2:**
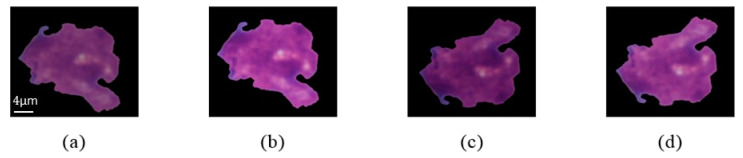
The result of applying data augmentation techniques (**a**) original image; (**b**) contrast adjustments and brightness correction; (**c**) vertical flip and contrast adjustments; (**d**) vertical flip, brightness correction, and contrast adjustments.

The performance of deep learning models may degrade due to imbalanced datasets or small numbers of training samples, where data dimensionality is much larger than the number of samples, or due to an imbalance in the number of training samples of each class. In order to avoid over-fitting of the classification networks, arising from small sized and/or imbalanced data, as suggested in [29], we employed different strategies of data augmentation, such as horizontal and vertical flips, contrast adjustments, and brightness correction to enlarge the dataset. Employing data augmentation techniques enables us to yield better performance compared to non-augmented data. However, geometric deformations, translation, reflection, rotation, and shear were not applied in order to preserve the texture and morphological properties of the original images. Some examples of ALL after the pre-processing and data augmentation steps are shown in Figure 2.

### 2.3. Feature Extraction and Architectures

#### 2.3.1. DCNN Models

Before going through the details of our proposed model, a brief introduction to the employed deep CNN architectures is presented in this section. The following architectures were selected for the first stage of this study.

The AlexNet [28] architecture won the ImageNet large-scale visual recognition challenge (ILSVRC) in the 2010 competition to classify 1.2 million images with 1000 categories. The basic architecture of AlexNet consists of five convolutional layers, two normalization layers, three max-pooling layers, three fully-connected layers, and a linear layer, with the softmax activation function in the last layer, and with 60 million parameters and 650,000 neurons. This architecture achieved a winning top-5 test error rate of 15.3%, compared to 26.2% achieved by the second-best entry.

VGGNet [19] was introduced by Karen Simonyan and Andrew Zisserman from the Visual Geometry Group (VGG) of University of Oxford in 2014 to examine the effect of the depth of the convolutional network on the final classification accuracy. This architecture achieved one of the top performances, with better feature extraction in the ILSVRC 2014 challenge, using a 3 × 3 convolution filter size, compared to AlexNet’s 11 × 11 convolution filter size. In this architecture, small filters increase the depth of the network instead of its width, which plays a critical role in gaining higher performance. There are two versions of this architecture: VGG16 and VGG19, with different depths and layers.

Neural architecture search net (NASNet) [30] achieved state-of-the-art results on the CIFAR10 dataset in 2017. Using a recurrent neural network, NASNet was able to search for the best convolutional layer in the CIFAR10 dataset, and then transferred it to the ImageNet dataset. Then more copies of this best convolutional layer were generated by stacking them together. However, this architecture is computationally expensive when the dataset is large. A new regularization technique called ScheduledDropPath was also introduced in this architecture to further improve generalization.

The Xception architecture, which stands for extreme inceptions consists of 36 convolutional layers, was made by François Chollet [20], and won the 2014 ILSVRC competition. Xception is able to extract discriminative features using multiple different filter sizes, such as 1 × 1, 3 × 3, and 5 × 5 in parallel. This architecture is inspired by Inception [31], wherein the Inception modules are replaced with depth-wise separable convolutions.

The densely connected convolutional networks (DenseNet) [32], proposed by Huang et al., are an extension of ResNet. In this architecture, layers are connected to each other by summation, leading to a better generalization and resilience to the problem of vanishing gradient. Feature maps of each layer can be reused as input for the next layers. The idea of reusing feature maps from preceding layers is beneficial for various computer vision tasks.

The InceptionV3 [18] architecture, proposed by Szegedy et al., won the ImageNet ILSVRC 2014 competition. It is a very deep network, consisting of 22 layers and having fewer parameters than AlexNet. The concept of the Inception module was introduced in this architecture. The Inception module is able to extract multi-level features in parallel, making it considerably more efficient and faster.

The MobileNet [33] architecture, proposed by Howard et al., is designed for mobile applications. The architecture consists of 28 layers, and is based on depth-wise separable convolution and a 1 × 1 point-wise convolution to extract discriminative features from input images. The architecture performance is evaluated on ImageNet classification and achieved an accuracy only slightly less than VGG16, while being 32 times smaller and 27 times less computationally intensive.

The ShuffleNet [34] architecture was proposed by the research team from Face++ (Megvii Inc.) in 2017 as an extremely computationally efficient deep CNN architecture. It was designed with fewer parameters, specifically for mobile devices with limited computation power. ShuffleNet uses point-wise group convolution and channel shuffle to speed up computation. With this reduction in the network size, there is no loss in accuracy when tested on ImageNet classification and MS COCO object detection, and it even performs slightly better (top-1 error (absolute 7.8%)) than MobileNet.

#### 2.3.2. Transfer Learning

Transfer learning is a common strategy in deep learning tasks, where a large dataset from a source task is used for training of a target task, leading to not only overcoming the problem of small datasets but also accelerating the learning process and improving the accuracy. Previous studies showed that transfer learning also has the potential to prevent over-fittings [35,36,37]. The transfer learning approach enables us to adopt a pre-trained network that has already learned a rich set of low-level features from layers that are closer to the input image. Though the dataset is not the same, the low-level features produced by the source CNN are mostly of general shapes, e.g., edges, contours, and curves, which are similar to the low-level features of the target dataset, while high-level features at the final layers concentrate on complex class-level characteristics, which are needed to differentiate between classes. With the use of transfer learning, training of large CNNs can now be a more practical strategy with more promising results, and significantly more cost-effective, by avoiding training a CNN from scratch.

#### 2.3.3. Local Binary Patterns

Local binary pattern (LBP) [38] is a texture feature descriptor which is able to extract local texture characteristics. In the literature, many researchers have attempted to utilize LBP feature descriptors as image texture descriptors for various image analysis applications [10,39,40] The main idea of the computation of LBP consists of two steps. First, the intensity value of each center pixel of a grey-scale image will be compared with each of the neighboring pixels or an interpolated sub-pixel location to generate a thresholded binary pattern. To construct the binary map, if the intensity value of the center pixel is greater or equal to its surrounding pixel, then it is given a value of 1, otherwise, 0. In the second step, the thresholded binary pattern is converted into its equivalent binary number. Mathematically, we can calculate the LBP value as follows:

(1) $$LBP\;\left(x_{c},y_{c}\right)=\;\overset{p-1}{\underset{p=0}{\sum }}ℱ\left(g_{p}-\;g_{c}\right)\;with\;ℱ\left(x\right)=\;\{\begin{array}{c} 1\;if\;g_{c}\;\geq \;g_{p}\\ 0\;otherwise \end{array}$$

In Equation (1), *xc* and *yc* are the image coordinates of a pixel. *gc* is the intensity of the pixel (*xc*, *yc*), and *gp* is the intensity of the *p*-th neighboring pixel. Function F is a binary thresholding function, which is applied to each of the surrounding eight local neighbor pixels to obtain the LBP map. Rotation invariance is achieved by circular permutation of the bits to obtain the largest possible integer.

### 2.4. Metrics for Performance Evaluation

To measure the prediction performance of the proposed method for this study, we computed common evaluation metrics, such as specificity, sensitivity, and classification accuracy. TP (true positive) is the number of positive instances that were correctly identified as positive; FN (false negative) is the number of positive instances that were incorrectly identified as negative. TN is the number of negative instances correctly predicted as negative, while FP is the number of negative instances incorrectly predicted as positive. Given TP, TN, FP, and FN, all evaluation metrics Equations (2)–(6) are based on the work presented in [16,22,29] were calculated as follows:

Sensitivity (also called recall or true positive rate (TPR)) is the proportion of positive (malignant) cases that were correctly classified:

(2) $$\mathrm{Sensitivity}\;\left(\mathrm{Recall}\right)=\frac{TP}{\left(TP+FN\right)}$$

Specificity is the proportion of negative (benign) cases that were correctly classified:

(3) $$\mathrm{Specificity}=\frac{TN}{\left(TN+FP\right)}$$

Precision is defined as the proportion of predicted positive instances (malignant) that were actually positive instances:

(4) $$\mathrm{Precision}=\frac{TP}{\left(TP+FP\right)}$$

Accuracy is the overall percentage of correctly classified instances:

(5) $$\mathrm{Accuracy}(\%)=\frac{TP+TN}{TP+TN+FP+FN}\times 100$$

F1 score, also known as F-measure, is defined as the harmonic mean of precision and recall:

(6) $$F-measure=2\times \frac{\left(precision\times recall\right)}{\left(precision+recall\right)}$$

## 3. Results

The implementation of our proposed model for ALL classification consists of the following steps: transfer learning using popular pre-trained DCNN architectures, hyper-parameter tuning of learning parameters, and ensemble technique in order to extract more discriminative features from a dataset. After dataset pre-processing, we employed various state-of-the-art deep CNN architectures, namely InceptionV3, AlexNet, DenseNet201, VGGNet-16, VGGNet19, Xception, MobileNet, ShuffleNet, and two NASNet models. All of the selected architectures have been used with success for classification problems in various medical image analysis tasks [35,36,41,42].

### 3.1. Experimental Dataset

This study was mainly based on a dataset of classification of normal versus malignant cells in B-ALL white blood cancer microscopic images (ISBI 2019) provided by SBI-Lab [11,12,43,44], which is available to the public at [21]. The goal of this challenge was to develop a machine learning solution for distinguishing normal cells from leukemic blast (malignant cells) in microscopic images of blood smears. All training and test images were stored in 24-bit RGB format, with a consistent size of 450 × 450 pixels. Illumination errors, inconsistent staining, and noises of the images were fixed with the methods provided by [11,12]. Regarding the proposed stain-normalization method, by using an SD-Layer, nine learnable parameters were included in two standard CNN models (AlexNet and Texture-CNN [45]). The proposed stain deconvolution layer (SD-Layer) is based on the optical density (OD) space. The size of the bounding box around a single cell is approximately 300 × 300 pixels. The images in this dataset were labelled as normal or malignant by an experienced oncologist. The training set contains a total of 76 individual subjects (47 ALL subjects and 29 Normal subjects), containing a total of 7272 ALL cell images and 3389 normal cell images. Given the current limitation of the dataset size, data augmentation techniques, such as contrast adjustment, brightness correction, and horizontal and vertical flips, enabled us to generate more than 70,000 images (as presented in Section 3.2). We tabulated the class distributions of the dataset before and after data augmentation in Table 1.

### 3.2. Experimental Networks Parameter Settings

For our experiments, 70% of the images of each class were assigned to the training set, 20% to the validation set, and the remaining 10% to the test set to evaluate the performance of different architectures. These splits were done randomly, and all sets were disjointed. Our experiment was implemented in Python [46] using the Keras [47] package, with Tensorflow [48] as the deep learning framework backend, and run on an Nvidia GeForce GTX 1080 Ti GPU with 11GB RAM. We examined the network hyperparameter configurations (e.g., depth of the network, gradient learning rate, the regularization parameter for ridge regression, weight initializations, batch size, learning rate, convolutional window sizes, dropout rate, regularization, and optimization strategy) using the grid-search technique, with different combinations of parameters, to obtain the best possible accuracies. We utilized ReLU with all layers, and dropout [49] in the last two fully-connected layers with a rate of 0.5, to prevent over-fitting. Dropout layers randomly eliminate some units’ connections to the next layer. The momentum term for the Stochastic gradient descent (SGD) optimizer was set to 0.7. β1 and β2 for Adam optimizer were set to 0.9 and 0.999, respectively. For the RMSProp optimizer, ρ was set to 0.7. Choosing a proper weight initialization method is also important to obtain optimal performance. For all of our networks, we initialized weights from weights trained on ImageNet [50]. We also applied L2 regularization on both the bias regularizer and kernel regularizer to regularize the weights of each layer, and avoid the overfitting issue. The L2 value for the bias regularizer and kernel regularizer were both set to 0.0001. For the fine-tuning of all models, we removed the final layer and froze the remaining pre-trained weights. Then, three fully-connected layers were added to all networks. For NASNetLarge, NASNetMobile, and InceptionV3, the batch size was set to eight and, for the rest of the networks, to 32, in order to satisfy GPU memory constraints. 50 epochs were used to train all models. For implementing the LBP texture descriptor, we used rotationally invariant uniform patterns, with radius 2 (R = 3) and 24 bits (N = 2). The extracted LBP histograms from each image were used as an input for the subsequent classification steps.

### 3.3. Study 1: Hand-Crafted Feature Extraction Based on Local Binary Patterns

For comparison purposes, classification results for LBP-based and DCNN-based features were compared. After extracting LBP histograms, the machine learning algorithms, including KNN [51], RF [52], gradient boosting decision tree (GBDT) [53], XGBoost [54], and naïve Bayes [55] using 5-fold cross-validation, were used to measure the performance of the classifiers. In this approach, first, LBP features were extracted for both the train and test set. Then, the extracted features were fed into the machine learning algorithms to predict the final output.

#### 3.3.1. Results on Local Binary Patterns

The obtained results for the selected models are presented in Table 2. The obtained results indicated that the XGBoost classifier achieved the best result, with 79.62% accuracy, and RF achieved the second-best result, with 77.96%.

Experiments for sensitivity, specificity, and precision metrics can be found in Tables S1–S3 in the supplementary file. 

### 3.4. Study 2: Deep Feature Extraction Based on Transfer Learning

The next experiment investigated the impact of normalization of the images using fine-tuned networks of AlexNet, NASNetLarge, NASNetMobile, DenseNet201, InceptionV3, VGG19, VGG16, Xception, MobileNet, and ShuffleNet. 

For the fine-tuning of these networks, we first pre-trained the networks on ImageNet data. In the network structures, convolution layers were followed by a max pooling layer. The last fully connected (FC) layer, which can be seen as a classification layer, of all selected pre-trained CNN networks was discarded. After that, we connected two new FC layers, each of which having 1024 hidden units with a rectified linear unit (ReLU) activation function. Finally, for the classification layer, two output neurons associating with normal and malignant cases, with a softmax nonlinear activation function, were used in the last layer. A specific example of our proposed network structure in the scheme of VGG19 can be found in supplementary file. 

#### 3.4.1. Results of Deep Feature Extraction Based on Transfer Learning

Table 3 shows the results obtained from different image normalization techniques. As can be seen, most of the models achieved the best results with dataset mean subtraction. MobileNet and DenseNet201 had better performance with ImageMean subtraction, and ImageNet mean subtraction techniques, respectively. Table 4 shows the accuracy obtained by different optimizers. Learners with the Adam optimizer had better performance compared to SGD and RMSProp in most of the models. The average of learners with Adam was roughly 2% better than SGD, and 10% better than RMSProp. To give some examples, DenseNet201 achieved 93.69% accuracy with Adam, which was a 1.83% accuracy gap with SGD, and a 2% accuracy gap with RMSProp. The MobileNet and ShuffleNet models had better performance with the SGD optimizer than with Adam, e.g., MobileNet achieved 92.45% accuracy with SGD, which had a 3.93% accuracy gap with Adam, and 0.21% accuracy gap with RMSProp. NASNetLarge and NASNetMobile had poor performance with the RMSProp optimizer.

### 3.5. Study 3: Deep Feature Extraction Based on Deep Learning Multi-Model Ensemble

Experiments for sensitivity and specificity of various normalization techniques can be found in Tables S4 and S5 in the supplementary file.

**Figure 3:**
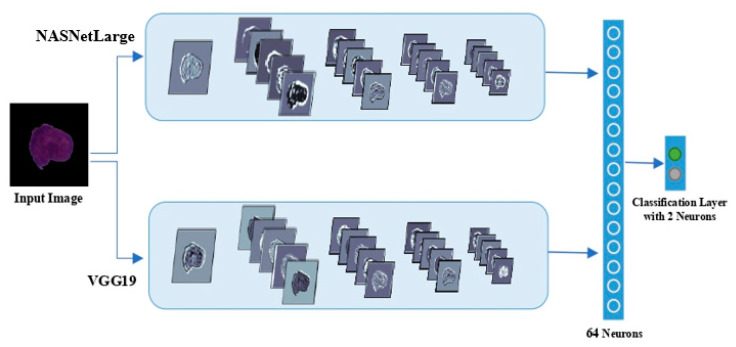
Illustration of the proposed aggregation-based architecture of VGG19 and NASNetLarge.

Various DCNN architectures with different parameters were selected for this experiment. However, only a few studies in the literature focused on the aggregation of multiple deep CNN models [41,56]. Hence, we were highly motivated to explore the potential of an ensemble model to apply more non-linearities, by connecting the trained weights of the best models obtained from study 2 to improve the final performance. The derived pre-trained weights from study 2 acted as initial parameters for the aggregated model. We selected architectures from study 2 based on their prediction performance. Then an aggregated-based learning strategy was employed to train the fused features extracted from the best models with appropriate fine-tuned parameters. Figure 3 shows a flowchart of a simplified illustration of the proposed DCNN ensemble architecture with VGG19 and NASNetLarge, i.e., the aggregated-based architecture of VGG19 and NASNetLarge as an example for this experiment. 

#### 3.5.1. Results of the Deep Learning Multi-Model Ensemble

Based on the results derived from Table 3 and Table 4 from study 2, the performance of the algorithms of NASNetLarge, VGG19, InceptionV3, and DenseNet201 achieved the best results using dataset normalization and the Adam optimizer; hence, we did not examine other parameters for all subsequent steps. Surprisingly, VGG19 achieved a good performance, despite being older and less sophisticated than the others. The results obtained from the various aggregated models are presented in Table 5 for ALL classification. From the table, it is clear that the aggregation-based deep models obtained better predictions than the individual models. 

**Figure 4:**
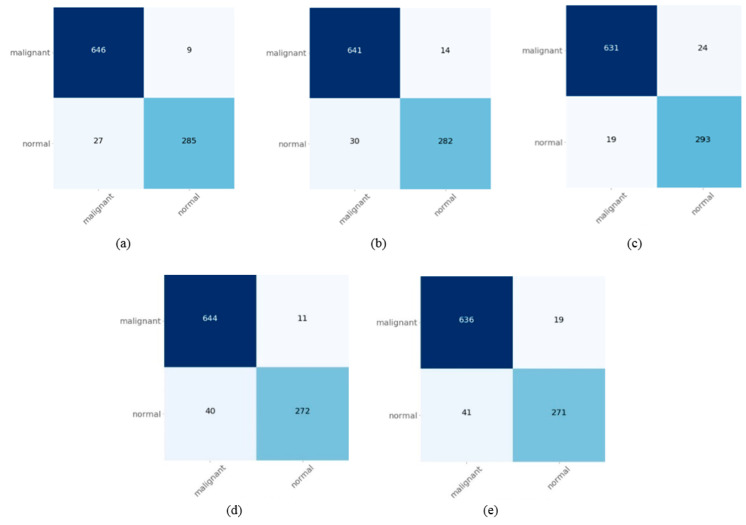
Confusion matrices obtained for different ensemble classifiers. (**a**) NASNetLarge and VGG19; (**b**) DenseNet201 and VGG19; (**c**) InceptionV3, VGG19, and DenseNet201; (**d**) DenseNet201 and InceptionV3; (**e**) InceptionV3 and VGG19.

Comparing the plain and aggregated architectures, the ensemble of NASNetLarge and VGG19 had more accurate detection rates (96.58%) than the individual NASNetLarge architecture (94.55%) and the individual VGG19 architecture (94.24%). The second-best architecture, the ensemble of DenseNet201 and VGG19, also yielded a higher overall accuracy (95.55%) than the individual DenseNet201 architecture (93.69%), and was slightly better than the individual VGG19 architecture (94.24%). The quantitative results for five ensemble models are also summarized in the form of confusion matrices in Figure 4. It can be seen that the aggregation of NASNetLarge and VGG19 architectures demonstrated better detection accuracy than the other ensembles. The misclassified cells of the NASNetLarge and VGG19 architectures were more commonly ALL cases that were mislabeled as normal cells. Intuitively, the main reason was that the morphology of some of the normal cells was more irregular and more typical of ALL cells. This may also be a matter of many cells being detected as FP. In this case, we tabulated the specificity value for all the combinations presented in Table 6. Referring to Table 6, the effect of false positive rates is reduced by balancing the distribution of classes on the dataset using data augmentation techniques. 

**Figure 5:**
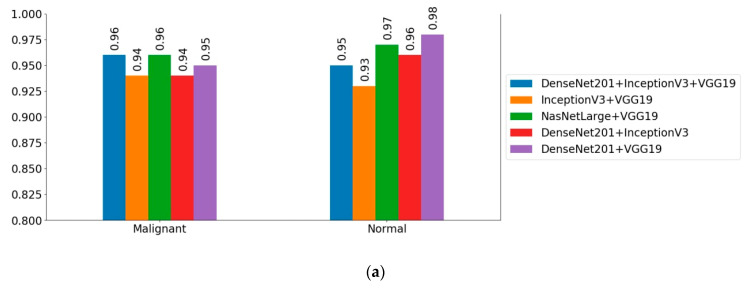

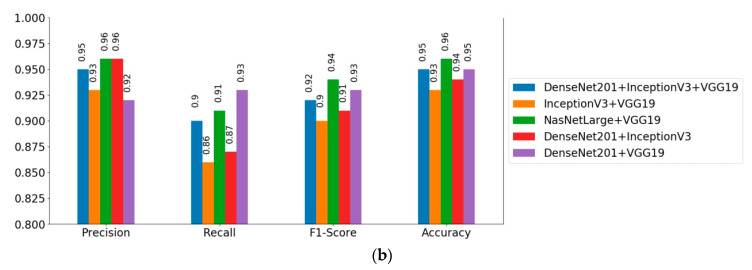
Confusion matrices obtained of different ensemble classifiers. (**a**) NASNetLarge and VGG19. (**b**) DenseNet201 and VGG19. (**c**) InceptionV3, VGG19 and DenseNet201. (**d**) DenseNet201 and InceptionV3. (**e**) InceptionV3 and VGG19.

Overall, the NASNet-Large with VGG19 was the best learner, and its counterpart, DenseNet201 with VGG19 was the second-best learner. A comparison of results of the aggregated models in ALL classification are presented in Figure 5. As an example, comparing the ensemble of NASNetLarge and VGG19 with the ensemble of InceptionV3 and VGG19, the former could gain a 4.78% gap in accuracy, 4.64% gap in F1-score, 4.89% gap in recall, and 3.49% gap in precision. Similar conclusions can be drawn for other ensemble models. In differentiating between malignant and normal classes in the test set, the ensemble of InceptionV3 and VGG19 had a poor result, with 94% for malignant cases and 93% for normal cases. The ensemble of NASNetLarge and VGG19 architectures had the best performance in detecting ALL cases, as demonstrated in Figure 5a.

## 4. Discussion

Based on the results shown in Table 5, we conclude that our proposed aggregated-base deep models can improve results in terms of accuracy, sensitivity, and specificity over other state-of-the-art algorithms in ALL prediction. In this experiment, widely adopted DCNN classifiers for medical image analysis were employed. All models were trained with 59,170 training images and validated on 18,384 images from the augmented dataset. The performance of the proposed method was evaluated with the 967 test images, comprised of 312 normal cases, and 655 ALL, which we did not use in the training and validation phase. We did not apply pre-processing and data augmentation steps to the test images. Different results were obtained from these classifiers based on their structure, characteristics, and hyper parameters. Based on the obtained results, we excluded those with lower accuracy, since classifiers with higher accuracy were able to extract more important features. We then implemented an ensemble model from the best algorithms to highlight the advantages of a multi-model classifier, with auxiliary supervision leading to a better prediction performance. The chosen ensemble architecture yielded a higher overall accuracy, of 96.58%, than the individual architectures. This behavior was expected because the NASNetLarge architecture is a recurrent neural network which enables transferability, and the ScheduledDropPath regularization technique aids the embedding of fine-grained information directly for feature extraction. Hence, the ensemble network was able to classify the unseen images more accurately. To show the effectiveness of our proposed method, the LBP hand-crafted features were used to extract features, and fed into various machine learning algorithms. On comparing the performance of the ensemble architectures, it was found that the aggregation of NASNetLarge and VGG19 had superior detection rates than the others. However, it should be emphasized that the computation time of the NASNetLarge architecture for each epoch during the training phase was approximately 130 min, in comparison with VGG19’s 20 min in GPU mode. Table 7 shows the number of parameters for each architecture in this study. We observed that the parameter number of NASNetLarge is quite large for training the network. Although the results by the proposed method are encouraging, there are some limitations of our method, highlighted as follows. First, large-scale datasets are needed in training deep learning applications, and the provided training data for this study was limited. To resolve this problem, various data augmentation strategies were employed. It would be more appropriate to have access to more reliable data sources by increasing the number of samples. Moreover, using pre-trained networks as feature extractors requires images to be resized to a certain dimension for some architectures, which may discard valuable discriminating information. In the future, we will focus on these points with the aim of reducing the false positive rate, and further improvement of the final accuracy. 

## 5. Conclusions

In this study, we presented an aggregation-based deep learning method for ALL classification, with an automation system to discern between healthy and cancer cases that strengthens the decision taken by the physician and reduces the workload. Another important aspect of our modeling was to greatly speed up the decisions for medical images to fractions of seconds (about 0.03 s per image, for the ensemble of NASNetLarge and VGG19) with a computer aided system. We analyzed various well-tuned deep learning architectures individually, as well as the proposed ensemble methods. Our proposed model could extract features from chosen pre-trained and fine-tuned deep CNNs. Extracted features were utilized as input to an ensemble deep neural network to obtain the final prediction. The obtained results from this study showed that our proposed deep learning-based model provides better discrimination ability in differentiating normal and ALL cells, outperforms the individual state-of-the-art networks, and yielded a higher overall accuracy, of 96.58%, than the individual architectures, as well as LBP-engineered feature extractor, and traditional machine learning, methods. We experimentally verified that the ensemble of NASNetLarge and VGG19 was the best model among all the learners. To resolve the problem of limited data size, we employed data augmentation techniques. A transfer learning strategy was also employed to accelerate the learning process, and further improve the performance of the proposed network. 

## Figures and Tables

**Table 1:** Total number of class samples before and after data augmentation.

Cell Type	Before Augmentation	After Augmentation
Healthy cells	3389	24,616
ALL cells	7272	52,938

**Table 2:** Accuracy (%) and standard deviation of different machine learning models over five-fold cross-validation of extracted LBP features. Each column in the table corresponds to the fold number. Bold values indicate the best result; underlined values represent the second-best result of the respective category.

Models	Fold1 (%)	Fold2 (%)	Fold3 (%)	Fold4 (%)	Fold5 (%)	Mean (%)	Std
KNN	78.86	89.69	89.11	74.61	55.44	77.54	± 0.13
Naive Bayes	55.67	85.05	66.83	90.67	79.27	75.50	± 0.12
Random Forest	76.28	88.65	92.22	78.23	54.40	77.96	± 0.13
Gradient Boosting	72.68	87.62	90.15	78.23	56.47	77.03	± 0.12
XGBoost	74.74	91.75	95.33	77.72	58.54	**79.62**	± 0.13

Bold values indicate the best result.

**Table 3:** Classification accuracies of various normalization techniques. In each row, the largest accuracy is shown in bold.

Models	Dataset Mean	Image Mean	Image Net Mean
AlexNet	**87.80%**	87.49%	86.45%
NASNetLarge	**94.55%**	91.73%	92.24%
NASNetMobile	**89.66%**	89.35%	87.18%
InceptionV3	**93.28%**	89.97%	92.14%
VGG19	**94.24%**	32.26%	91.31%
VGG16	**93.28%**	67.74%	86.35%
Xception	**92.97%**	91.11%	92.14%
MobileNet	88.52%	**90.49%**	87.90%
ShuffleNet	**80.35%**	80.04%	77.56%
DenseNet201	93.69%	92.76%	**94.93%**

Bold values indicate the largest accuracy.

**Table 4:** Classification accuracies of Adam, SGD, and RMSProp optimizers. In each row, the largest accuracy is shown in bold.

Models	Adam	SGD	RMSProp
AlexNet	87.80%	85.73%	**88.11%**
NASNetLarge	**95.55%**	92.14%	32.26%
DenseNet201	**93.69%**	91.86%	91.68%
NASNetMobile	**89.66%**	88.93%	60.70%
InceptionV3	**93.28%**	92.66%	92.86%
VGG19	**95.24%**	88.24%	93.90%
VGG16	**93.10%**	90.49%	91.49%
Xception	**92.97%**	88.13%	91.31%
MobileNet	88.52%	**92.45%**	92.24%
ShuffleNet	80.35%	**84.49%**	80.66%
Average	**90.82%**	89.25%	81.52%

Bold values indicate the largest accuracy.

**Table 5:** Classification results of different architectures using the proposed ensemble method. We selected architectures from study 2 based on their prediction performance. The measures used to quantify the quality of the results were the accuracy, precision, recall, and F-measure.

Ensemble Models	Accuracy (%)	Precision (%)	Recall (%)	F1-score (%)
NASNetLarge + VGG19 *	96.58 ± 1.09	96.94	91.75	94.67
DenseNet201 + InceptionV3	94.73 ± 1.27	96.11	87.18	91.43
DenseNet201 + VGG19 ^$^	95.55 ± 1.10	92.43	93.91	93.16
InceptionV3 + VGG19	93.80 ± 1.31	93.45	86.86	90.03
DenseNet201 + VGG19 + InceptionV3	95.45 ± 1.29	95.30	90.38	92.76

* The best result and ^$^ the second-best result.

**Table 6:** Sensitivity and specificity of different architectures using the proposed ensemble method. Bold values indicate the best accuracy result.

Models	Sensitivity (%)	Specificity (%)
NASNetLarge	94.45	94.13
DenseNet201	92.89	94.90
VGG19	96.24	91.88
InceptionV3	92.53	91.43
DenseNet201 + VGG19 + InceptionV3	95.52	95.27
NASNetLarge + VGG19	95.98	96.93
DenseNet201 + InceptionV3	94.15	96.11
DenseNet201 + VGG19	97.07	92.42
InceptionV3 + VGG19	93.94	93.44

**Table 7:** Total number of parameters of DCNN models.

Model	Total Parameters
NASNetLarge	90 M
InceptionV3	24 M
AlexNet	22 M
DenseNet201	21 M
VGG19	21 M
Xception	20 M
VGG16	16 M
NASNetMobile	6 M
MobileNet	5 M
ShuffleNet	1 M

## Data Availability

The data used in this work are from public datasets: ISBI 2019 C-NMC Challenge: Classification in Cancer Cell Imaging (https://competitions.codalab.org/competitions/20395). To apply for the access to dataset, a registration is required.

## Supplementary Materials

The following are available online at https://www.mdpi.com/2075-4418/10/12/1064/s1.
