# Supplementary material for: An Aggregated-Based Deep Learning Method for Leukemic B-lymphoblast Classification
Source: Diagnostics (Basel). 2020 Dec 8;10(12):1064. doi: 10.3390/diagnostics10121064 (PMC7763941; doi:10.3390/diagnostics10121064)
Supplement: Supplementary file 1 [file diagnostics-10-01064-s001.pdf]

Supplementary files

We bring details of our experiment with additional information and many other important metrics related to the classification task.

Tables S.1 and S.2 and S.3 tabulate the sensitivity, specificity and precision of different machine learning models with extracted LBF features. Referring to Tables, XGBoost is best classifier with better average of sensitivity and specificity and precision compared to other learners. The second-best learner is KNN in terms of sensitivity and Random Forest in terms of Specificity metric.

Table S.1. Sensitivity (%) and standard deviation of different machine learning models over 5 folds cross-validation of extracted LBP features. Each column in the table corresponds to the fold number. Bold values indicate the best result; underlined values represent the second-best result of the respective category.

Models	Fold1 (%)	Fold2 (%)	Fold3 (%)	Fold4 (%)	Fold5 (%)	Mean (%)	Std
KNN	73.63	86.02	84.03	76.37	55.37	<u>75.08</u>	± 12.6
Naive Bayes	56.83	83.74	69.84	84.12	73.62	73.63	± 11.3
Random Forest	70.22	80.06	87.20	79.27	55.52	74.45	± 12.8
Gradient Boosting	69.92	85.70	88.28	76.32	52.45	74.53	± 14.3
XGBoost	72.01	88.11	90.12	79.66	57.57	**77.49**	± 13.2

Table S.2. Specificity (%) and standard deviation of different machine learning models over 5 folds cross-validation of extracted LBP features. Each column in the table corresponds to the fold number. Bold values indicate the best result; underlined values represent the second-best result of the respective category.

Models	Fold1 (%)	Fold2 (%)	Fold3 (%)	Fold4 (%)	Fold5 (%)	Mean (%)	Std
KNN	66.93	83.10	86.30	75.35	56.41	73.62	± 12.19
Naive Bayes	53.36	81.24	66.85	88.35	73.34	72.63	± 13.48
Random Forest	75.22	85.63	91.02	73.53	54.43	<u>75.97</u>	± 14.06
Gradient Boosting	70.09	82.68	89.11	72.48	53.42	73.56	± 13.64
XGBoost	72.27	88.74	92.37	75.20	54.47	**76.61**	± 15.05

Table S.3. Precision (%) and standard deviation of different machine learning models over 5 folds cross-validation of extracted LBP features. Each column in the table corresponds to the fold number. Bold values indicate the best result; underlined values represent the second-best result of the respective category.

Models	Fold1 (%)	Fold2 (%)	Fold3 (%)	Fold4 (%)	Fold5 (%)	Mean (%)	Std
KNN	67.37	85.38	84.24	75.57	56.42	73.80	± 12.14
Naive Bayes	54.63	82.37	66.56	86.52	73.37	72.69	± 12.74
Random Forest	76.09	88.92	92.67	78.63	58.45	**78.95**	± 13.38
Gradient Boosting	72.67	81.11	88.56	74.72	54.55	74.32	± 12.68
XGBoost	75.26	86.32	92.63	77.24	54.40	<u>77.17</u>	± 14.53

We also illustrate the schematic of the proposed network in Figure 3 with VGGNet architecture as an example in Figure S.1. This helps to better understand the structure of our proposed learners.

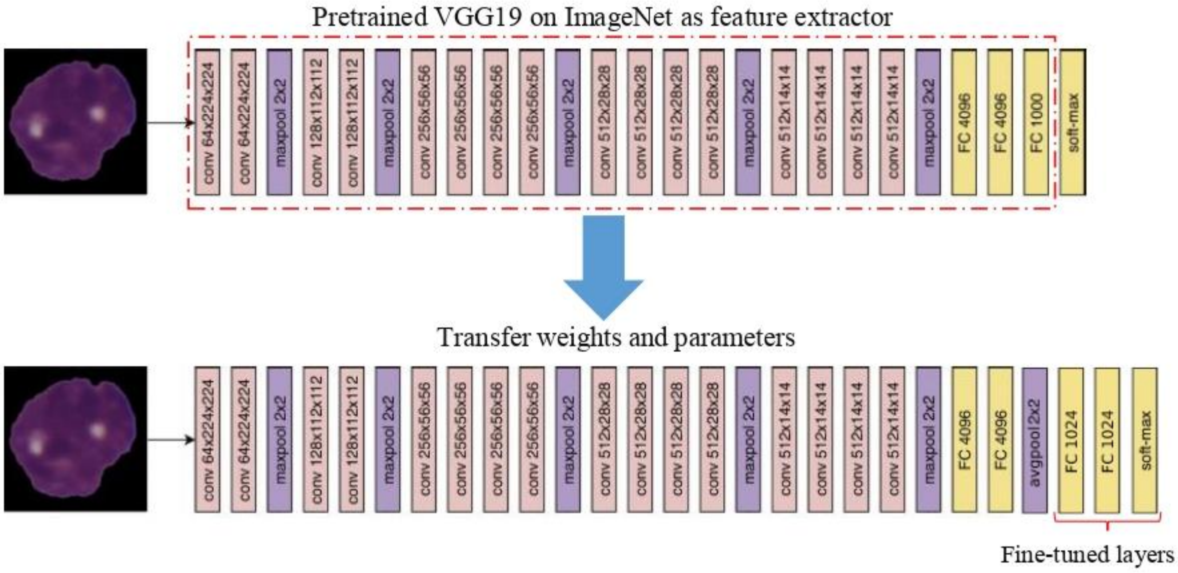

Figure S.1. Illustrations of fine-tuned VGG19 architecture. The final FC layer of the original architecture with 1000 classes is replaced by two FC layers and a final softmax layer as classification layer.

Tables S.4 and S.5 show the results of sensitivity and specificity obtained from different optimizers. Learners with Adam optimizer have better performance compared to SGD and RMSProp in most of the models.

Table S.4. Sensitivity of various normalization techniques. In each row, the largest accuracy is shown in bold.

**Models**	**Adam**	**SGD**	**RMSProp**
AlexNet	**86.42**%	84.73%	86.11%
NASNetLarge	**94.25%**	92.24%	39.26%
DenseNet201	**92.63%**	91.86%	90.68%
NASNetMobile	**89.68%**	88.93%	62.70%
InceptionV3	**92.22%**	91.66%	91.86%
VGG19	**94.53%**	87.24%	92.90%
VGG16	**92.92%**	90.49%	90.49%
Xception	**92.19%**	86.13%	90.31%
MobileNet	85.24%	**91.45%**	91.24%
ShuffleNet	80.41%	**83.49%**	82.66%
Average	**90.08%**	88.83%	81.82%

Table S.5. Specificity of various normalization techniques. In each row, the largest accuracy is shown in bold.

**Models**	**Adam**	**SGD**	**RMSProp**
AlexNet	**85.80**%	83.73%	85.11%
NASNetLarge	**94.55%**	90.14%	35.26%
DenseNet201	**91.69%**	89.86%	90.68%
NASNetMobile	85.66%	**86.93**%	61.70%

InceptionV3	**92.28%**	90.66%	90.86%
VGG19	92.24**%**	83.24%	**92.90**%
VGG16	**90.60%**	86.49%	90.49%
Xception	**90.97%**	85.13%	90.31%
MobileNet	85.52%	**91.45%**	90.24%
ShuffleNet	80.35%	**83.49%**	77.66%
Average	**88.94%**	87.11%	80.52%
